# Study on the Characteristics of Molten Glass in a Float Glass Process with a New Structure

**DOI:** 10.3390/ma17204989

**Published:** 2024-10-12

**Authors:** Benjun Cheng, Hao Feng, Feng Wu, Xiaocheng Liang, Mao Li

**Affiliations:** 1 School of Energy Science and Engineering, Central South University, Changsha 410083, China; chbj666@csu.edu.cn (B.C.); 223912041@csu.edu.cn (H.F.); 2 School of Materials and Metallurgy, University of Science and Technology Liaoning, Anshan 114054, China; wufeng@ustl.edu.cn

**Keywords:** float glass process, glass furnace, numerical simulation, thermal, flow characteristic

## Abstract

Glass is one of the most common materials in society, and the float glass process is the main production method of glass used at present, which involves adopting a melting furnace with a single cooler. However, this structure has been difficult to fit to the requirements of modern glass production, such as producing multiple types of glass and large-scale production. Therefore, a large-tonnage float glass melting furnace with a double cooler is studied, which is rising in popularity in the glass sector. The aim of this paper is to clarify the characteristics of the new glass furnace. A numerical simulation technique is applied to analyze the thermal and flow characteristics of molten glass in the new structure so as to clarify the feasibility of production by checking the temperature distribution and flow field of the molten glass. The results show that the new structure also exhibits flow behavior similar to the original structure in the branch line. Due to the addition of the branch line, the stability of the temperature is improved, with a 60 K and 43 K difference between the surface and bottom in the main and branch lines, respectively. Similar stability is shown in the flow field, specifically low acceleration in the cooler (0.006 m/s^2^). The bubble clarification time is about 2700 s, less than the 3000 s required for flow. The parameters of the branch line meet the requirements of glass production. In theory, a glass-melting furnace with a double cooler has the capacity to produce two types of glass.

## 1. Introduction

**Figure 1:**
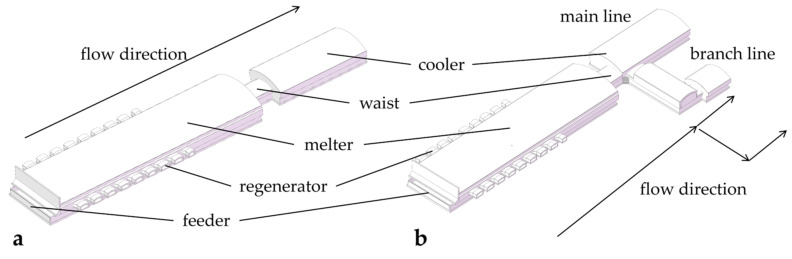
Float glass melting furnace; (**a**) single cooler and (**b**) double cooler.

Glass, recognized as a pivotal building material, has been extensively utilized in contemporary society. Its historical lineage can be traced back approximately 4000 years, and subsequent extensive development has contributed to the complexity of glass composition and its versatile applications [1]. In addition to sodium calcium glass, mainly composed of Na_2_SiO_3_ and CaSiO_3_, there is also tempered glass, colored glass, potassium glass, and microcrystalline glass [2,3,4,5,6]. Almost all glass production has been related to glass furnaces since the introduction of the float glass process in 1953, which is now the main production process for glass. This process utilizes the tension between the molten glass and the molten metal so that the molten glass spreads out on the surface of the molten metal. Consequently, the process facilitates the attainment of crucial properties, such as flatness, homogeneity, and the preservation of the glass’s inherent purity. The float glass-melting furnace serves as the central equipment in float glass technology, a typical structure for which is shown in Figure 1. As the core equipment in the float glass process, the glass melting furnace includes a regenerator, feeder, melter, cooler, and outlet. The regenerator provides heat to the melter, where raw materials melt, clarify, and homogenize. Then, the molten glass is cooled until it reaches the required temperature for forming and finally flows out of the outlet [7]. Along with the furnace with single cooler’s contributions to the industry, people have also discovered its drawbacks, such as its low production (≤1000 tons per day), few types of production, and that the produced glass is not thin enough. Thus, scholars raise a new structure with more coolers in one furnace: the glass melting furnace with a double cooler. It is developing rapidly, given its capacity to produce multiple products in one furnace, although its stability during large-tonnage production and the complexity of related working conditions are still not clear. It is essential to study the float glass melting furnace with a double cooler.

The float glass process has made significant progress in its application. Research about float glass processes is concentrated on the refractory of furnace walls; the working parameters and components of glass, particularly with respect to whether the furnace is ruined; and additional processes and additives [2,3,8,9]. Here, we refer to studies about numerical simulation. In terms of modeling, a mathematical model for hydrodynamics in glass-melting furnaces was established, which could well describe the actual production of 300 tons per day. Researchers applied this model to the horseshoe glass melting furnace, a shape different from the original glass melting furnace, to study flame and heat transfer, proving that numerical simulation can guide the production of float glass to a certain extent [10,11,12]. Other researchers used the Euler model to describe the pile, molten glass, and bubbles in a glass melting furnace, thereby establishing a glass furnace model (GFM) that includes combustion space and molten glass, which has the ability to study commercial-scale, single-cooler float glass furnaces [13]. Process parameters and components of glass are conducted using numerical simulation on single-cooler glass melting furnaces, such as homogenization, flow rate, and the daily feed rate of molten glass. Those studies clarified the behavior of molten glass inside the single-cooler melting furnace and provided a large number of optimized single-process parameters [14,15,16]. The synergistic effect of melting and flow was studied by combining heat transfer and conversion kinetics in a model, which pointed out that the temperature history of raw materials particles plays a crucial role in the melting process when considering conversion kinetics [17]. A similar method was used in the effect of the cold cap in an electric melter [18]. In recent years, scholars have focused more on active behavior in furnaces to improve production efficiency. They declared that physical firing methods could enhance the quality of glass and energy efficiency, which reminded manufacturers of an interesting way to optimize the float process.

The flame space, which provides heat to molten glass, is also a hot topic in the field. Gas or oil are always used for fuel, reacting with oxygen in the air or full oxygen, and their combustion contains strong heat and mass transfer. To reach higher economic efficiency, researchers focused on inhomogeneities, emissions, energy saving, airflow organizing, and electric coupling [19,20,21] with a furnace production of 300–600 tons per day. Those studies all announced that premixed fuel could boost the combustion and raise the melting temperature. At the same time, some of them pointed out that expanding the burner and exhaust gas outlet could improve the organization of airflow and prolong the gas resistance time in the furnace.

Except for process parameters, the researchers also highlight the structure of the glass melting furnace. Y. Wang [22] developed a three-dimensional melting furnace model, which describes the effect of variables such as geometry on the heat load of the furnace, taking into account the heating of the flame-burning space. In a single float glass melting furnace, there are some special parts that are important; for example, the spout lip and electrode positions have been studied. Dependent on these studies, when the molten glass flows out of the spout lip, the reflux at the edge is large, which facilitates the concentration of impurity-containing molten glass to the edge, thus improving the quality of glass plates [23]. And a method for assessing the melting efficiency and product quality within the critical particle mass index is proposed, which promotes the application of clean energy in the float process [24]. Glass quality analysis is always difficult to combine with glass melting simulation, not only as given above but also in energy efficiency, as included by Juraj Raic et al. [25]. In addition to parameters that are indispensable in normal conditions, they suggested other indices like the melting index and sand dissolution index to evaluate glass quality and energy efficiency during the melting process.

In terms of both process and structure, researchers have always based their work on a single cooler. In particular, R. Chen et al. [26] proposed melting glass in a crucible by induction heating, which is an interesting proposal: induction heating has the advantages of melting quickly and being energy-efficient; shortening the melting cycle may compensate for the discontinuity caused by crucible production. Even though this idea has not yet achieved reliability in large-scale production, some manufacturers have recently tried to apply the double cooler glass melting furnace reformed by the single cooler in order to increase the variety of products.

Upon reviewing the available literature, most of the studies have not contributed to a new structure to revolutionize the production of glass yet. As a new development, a float glass melting furnace with a double cooler (FFDC) has now been designed in manufacturing, which is reformed by a single cooler in order to boost the variety of products and, further, to apply in the large-tonnage furnace. However, systematic work has not been conducted on this new structure and on large-tonnage furnaces. Therefore, the study of the float glass process with the double cooler structure based on numerical simulation is a new attempt. After all, there are plenty of differences between single and double coolers; some operating parameters and optimization methods are yet to be clarified. Currently, in real production, this new design has indicated better convenience, better clarification, and lower energy consumption than the traditional structure, and it creatively puts forward a direction for glass sector improvement. The temperature distribution and liquid glass flow characteristics are studied in this paper, and based on it, plenty of methods which are used in past studies could pave the way for improving new structures of glass production.

## 2. Model Description

### 2.1. Physical Model

**Figure 2:**
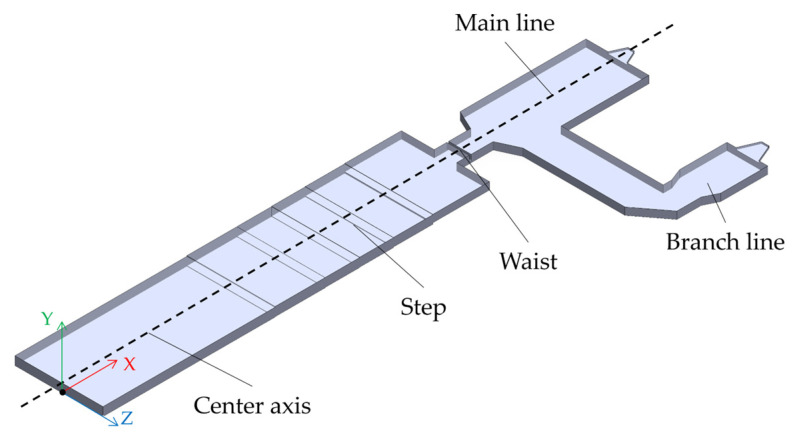
Liquid field of molten glass.

**Figure 3:**
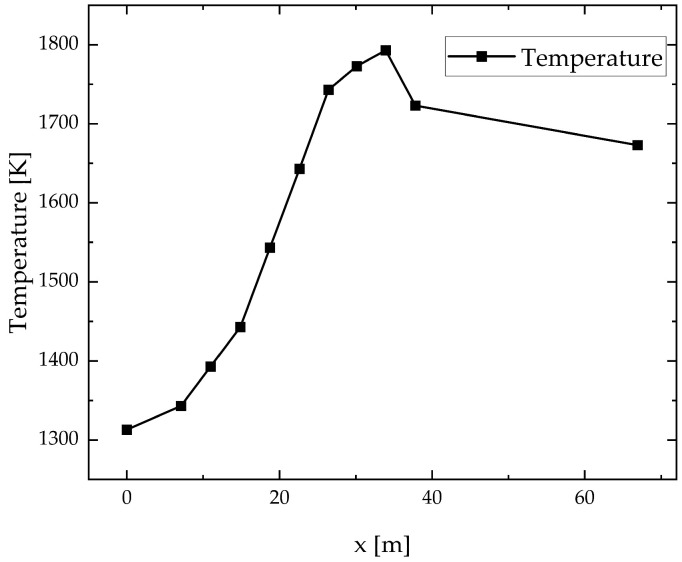
Boundary condition of temperature.

Stemming from a 1300 tons per day actual FFDC in service at a glass production company in Hubei Province, China, a model of the molten glass, as shown in Figure 2, has been developed by selecting molten glass for the object of study. The interface between the pile and the molten glass is chosen for the velocity inlet, with a velocity of 1.59 × 10^−5^ m/s based on the daily production rate. The equivalent hydraulic diameter of the channel is 18.49 m. The heat transfer coefficient is 5 W/m^2^∙K on the upper surface, and on the other three surfaces is 3.5 W/m^2^∙K by measuring the real melting furnace. Because the heat comes from the flame space above the molten glass, the boundary condition of temperature is measured by the surface of the actual melter and compiled by UDF (user-defined functions), which is shown in Figure 3. Molten glass flows through the waist and double cooler and finally out of the outlet, which is defined in the outflow. The major size of the furnace model is shown in Table 1. The physical parameters of molten glass via experiments and comparisons with other works [27,28] are shown in Table 2.

### 2.2. Mathematical Model

**Figure 4:**
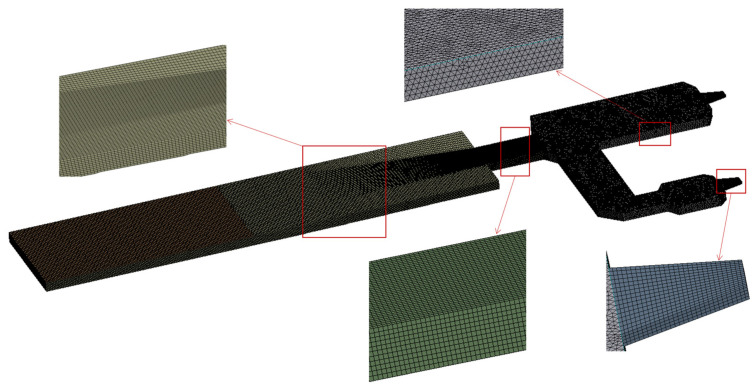
Global grids.

The behavior of molten glass in the melting furnace follows the laws of conservation of mass, momentum, and energy. The mathematical expressions corresponding to these principles in hydrodynamics are the continuity equation, the momentum conservation equation, and the energy conservation equation, respectively. According to the Reynolds number, the flow inside the glass melting furnace should belong to laminar flow, which has also been supported in similar studies [14,15,16]. The global grid size is 0.2 m due to the size of the outlets and waist, and the grids in these locations have been refined by 0.1 m. The total grid number is 1,019,051, and the grid type is hexahedronal. The grids and local details, which are indicated by arrows and squares, are shown in Figure 4.

#### 2.2.1. The Continuity Equation

The continuity equation is an expression of the conservation of mass for a moving fluid. For a moving space filled with molten glass, a system with fixed boundaries is taken as the control body, and the volume and area of the control body do not change with time.

In a three-dimensional Cartesian coordinate system, the continuity equation of molten glass is shown as Equation (1).

∂ρ/∂τ + (∂(ρV_x_))/∂x + (∂(ρV_y_))/∂y + (∂(ρV_z_))/∂z = 0 (1)

where ρ is the density of molten glass, τ is time, V_x_, V_y_, and V_z_ are the velocity components of the molten glass in the x-, y-, and z-directions, respectively.

#### 2.2.2. The Navier–Stokes Equations

In computational fluid dynamics, Navier–Stokes equations (N-S equations) are used to describe the change in momentum. The fluid element in motion is subjected to two forces: volume force and surface force. Volume force is the force directly acting on the entire element of the particle, and surface force is the force directly acting on the surface of the particle; they are manifested as gravity and pressure, respectively. The change in momentum comes from the combined effect of these two forces. Because of difficulties in solving N-S equations, the irrotational flow is applied in this model, thus obtaining analytical solutions of glass flow. In the three-dimensional coordinate system, the specific mathematical description is shown in Equation (2).

(∂ρV_i_)/∂t + ∂(ρV_x_V_i_)/∂x + ∂(ρV_y_V_i_)/∂y + ∂(ρV_z_V_i_)/∂z = ρg_i_ − ∂P/∂i + R_i_ + ∂/∂x (μ (∂V_i_)/∂x) + ∂/∂y(μ_e_(∂V_i_)/∂y) + ∂/∂z (μ_e_(∂V_i_)/∂z) (2)

where g is the gravity component in the i-direction, μ_e_ is the dynamic viscosity, P is the absolute pressure, R is the distributed resistance, the subscript i represents direction, and i = x, y, z.

#### 2.2.3. The Energy Equation

The energy equation is used to describe the conservation of energy as density, temperature, and internal energy change. The energy equation can be expressed as the rate of increase in thermodynamic energy in an element equal to the net heat flux into the element and the sum of the work carried out on the microcosm by the volumetric and surface forces [29]. This is specifically described as shown in Equation (3).

∂/∂t(ρE) + ∇ [u(ρE + ρ)] = ∇ [k_dff_∇T − ∑_j_[h_j_·J_j_ + (τ_eff_·u)] + S_h_ (3)

where E is the total kinetic of the fluid element, h_j_ is the enthalpy of component j, k_dff_ is the effective thermal conductivity, J_j_ is the diffusion flux of component j, and S_h_ is the source term of volumetric heat.

### 2.3. Numerical Solution Technique

ANSYS Fluent 18.0 is utilized as the simulation software for solving the problem. The ANSYS Mesh module is employed to create the mesh for the simulation, and CFD-Post is used for postprocessing. The coupled method is chosen as the solution method; the flux type is distance-based; and spatial discretization parameters are default. Relaxation factors are default as well. A standard initialization method is selected for initialization. The convergence criterion for the solution equation is set to a residual value of 1 × 10^−5^. The physical model is founded by Solidworks 2020.

### 2.4. Mesh Independence Verification

The meshes are refined in the x, y, and z directions by ±10% to verify that the simulation results are dependent on the size of the mesh. The maximum temperature, minimum temperature, location of vortexes, and shape of the flow should closely match with the original results, with a quantitative error within 2%. The data of verification are shown in Table 3.

## 3. Results and Analysis

### 3.1. Temperature Distribution

**Figure 5:**
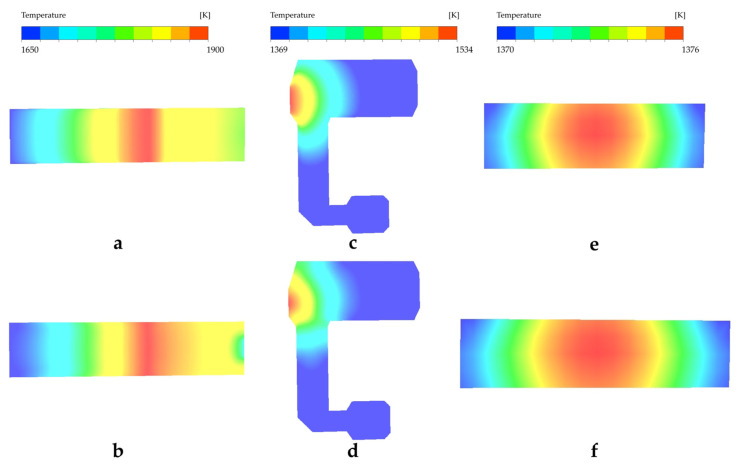
Temperature distribution: (**a**) surface of melter, (**b**) bottom of melter, (**c**) surface of cooler, (**d**) bottom of cooler, (**e**) outlet of main line, and (**f**) outlet of branch line.

The temperature distribution is shown in Figure 5, where (a) and (b) show a high trend in the middle and low parts at both sides in the temperature of the melter, which is caused by the arrangement of the furnace, i.e., higher heat load in the middle and lower parts in both ends. Currently, there is no better temperature distribution pointed out by any studies from the literature, but in production, the location of maximum temperature, which is called a hot spot, is always located in 1/2 to 2/3 of the whole melting section in the direction of flow. The maximum temperature occurs at about 32.5 m far from the face wall, which is at 54.7% of the melting section and fits the requirement. At the bottom of molten glass, a lower and smoother temperature distribution is represented than the surface; this is caused by the flame space heating the surface, and the heat transfers to the bottom mainly through the heat conduction of molten glass. At the end of the melter, there are a few differences between the surface and bottom because of the waist. Figure 5c,d indicate that the temperature distribution of the cooler is affected by heat dissipation and heat from the waist; the highest temperature occurs at the inlet of the cooler. Unlike the single cooler, because of the existence of a branch line, the temperature reveals radial distribution in the shape of a half-circle; the max temperature reaches 1530 K (1257 °C), which is at the waist. The temperature differences in the surface and bottom at the main line and branch line are 60 K and 43 K, respectively, which indicates better stability after one. Combined with Figure 5e,f, the temperature differences between the inlet and outlet of the main and branch lines are 162 K and 189 K on average. At the same time, a radial temperature distribution appears in the outlet of two lines, with a 3 K difference between the max and min temperature of each line outlet and a 4 K average between the two lines. The reason for the larger temperature difference in the branch line is that the flow path of the branch is longer than the main line, which gives enough time to cool the molten glass through natural heat dissipation. The other potential reason is presumed to be that a greater diversion of the branch lines causes a higher convective heat transfer coefficient [30].

### 3.2. Flow Field

**Figure 6:**
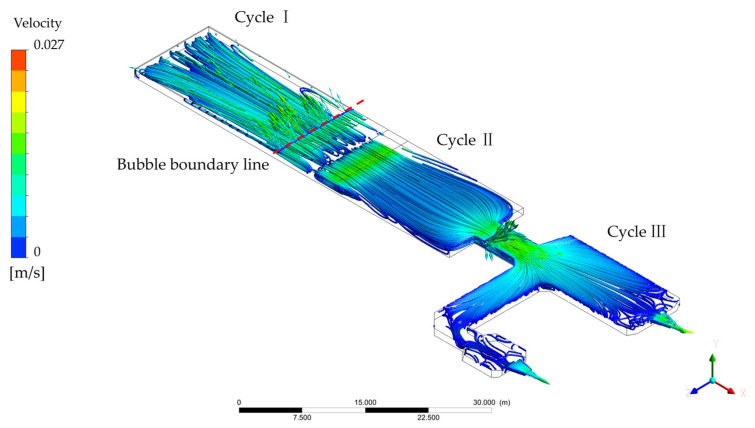
The streamline of molten glass in FFDC.

**Figure 7:**
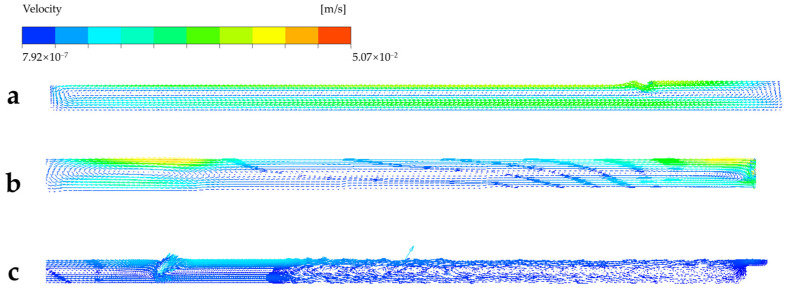
Vortex of XZ plane (**a**) Cycle I (**b**) Cycle II (**c**) Cycle III.

The flow field is shown in Figure 6 and Figure 7. Figure 6 indicates the streamline of the whole FFDC. It can be seen that the reduction gives rise to the velocity gradually increasing at the head of the melter; the trend stops at about 27 m away from the inlet face wall. This is called bubble boundary in production as it has bubbles on the molten glass from the pile. The maximum velocity reaches 0.027 m/s located here. The velocity of glass is one of the most important parameters in the float glass process because it is closely linked with the quality of the glass forming; it could cause irregularity, turbidity, and a block by being too fast or too slow. Considering the clarification, for example, the above simulation result indicated that the average residence time of the glass after the bubble line to the waist is about 3000 s. From Stokes’ law [7], as given in Equation (4), the average temperature of the liquid glass is about 1595 K. From the aforementioned density–temperature linear relationship ρ(T) and kinetic viscosity–temperature linear relationship μ(T), with the assumption that the diameter of the air bubbles D = 0.001 m, the uplift velocity is about 6 × 10^−4^ mm/s. So, the time required for bubbles to rise from the bottom to the top is about 2700 s. Therefore, it can be further assumed that the majority of air bubbles can reach the liquid surface and exit before the liquid glass reaches the waist.

v_f_ = 2000rg·Δρ/(9μ) (4)

where v_f_ is the velocity of bubble lift, r is the equivalent diameter of the bubble, Δρ is the difference density between the liquid glass and gas in a bubble.

After the bubble boundary, because of several sudden enlargements, velocity decreases in this area until it is next to the waist. Waist is a reducing structure in a glass melting furnace that causes the velocity of molten glass to increase quickly. Molten glass flows across the waist and is divided into two, the main and branch line, in the cooler. In the main line, it can be found that in Figure 6, a part of the molten glass spreads to the whole main line, and reflux occurs in front of the cooler wall; the other part flows in the branch line and has the same reflux. The acceleration is about 0.006 m/s^2^ in both coolers. Something different is that reflux is acted out more frequently than the main line because of more blocks in the channel of the branch line. We infer from this that the effect of cooling in the branch line is better than the main line. In fact, the average temperature in the main and branch lines is 1449 K (1176 °C) and 1366 K (1093 °C), respectively, which supports this speculation. The comparison with other studies that stem from the single cooler in Figure 8 of Ref. [16] indicated that, in the melting section, similar characteristics are shown. The streamline of the molten glass clearly divides into two parts by the bubble boundary line, and their size depends on the location of the bubble boundary. Divergence is caused after the waist. FFDC makes the molten glass partly flow into the branch line; thus, the streamline creates more chaos than a single cooler. This phenomenon creates the possibility of clarifying glass in the cooler continuously. In addition, the velocity of the cooler in both structures is the same at about 0.01 m/s, which shows that the turbulence in FFDC develops an advantage in glass production.

**Figure 8:**
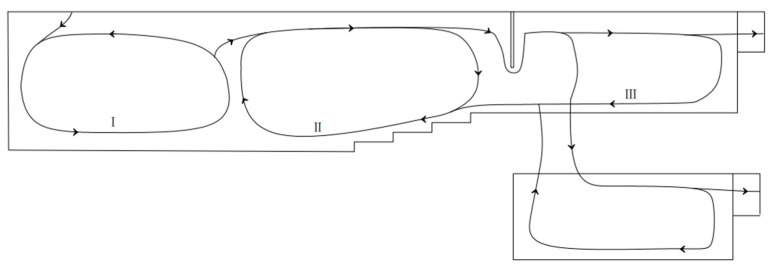
The trajectory of molten glass in FFDC.

Three flow cycles are referred to in some research on float glass melting furnaces with single lines, and they have proven to play a crucial role in glass production [14,30]. In the new structure, the three flow cycles similar to a single line are discovered and shown in Figure 6. The formation of cycles is mainly influenced by three factors: difference in molten glass density caused by different temperatures, compensation of molten glass flowed out, and block out by furnace wall. We continued to use the names Cycles I, II, and III. Cycle I is located in the head of the melter; here, the pile absorbs heat because the reaction causes the molten glass to cool down; thus, density increases, and molten glass sinks and flows ahead along the bottom. Because of the step of the bottom, molten glass belonging to Cycle I is forced to flow up. At the same time, the upper molten glass is blocked by the waist and refluxing. Finally, Cycle II takes its shape. Paradoxically, however, lower molten glass must flow out from the melter to the cooler via the space, which is located at the bottom of the channel. To find a new harmony, part of the molten glass separates from the reflux and flows into the cooler. When molten glass flows in the cooler, it spreads surroundings and is blocked by a wall of the cooler, which establishes Cycle III. Cycle III appears in both the main and branch lines simultaneously, which is different. The flow is more chaotic because of more twists in the branch line. The three flow cycles in FFDC are shown in Figure 8.

## 4. Discussion

FFDC, as a new structure in the float glass process, is applied in some places. Although its product seemingly has advantages now, there are plenty of uncertain factors because it will be applied soon. Firstly, FFDC has a second additional cooler unlike the traditional structure; it inescapably causes more construction space and more lining refractory materials. Even though the extra cost should be computed with the glass furnace’s long lifetime of more than 80 years, its economic efficiency is worth considering. Subsequently, the new structure is more complex than the traditional one, and malfunctions and maintenance are new challenges, too. Finally, the sector of glass production, where the highest temperature is located is called the hot spot. The two parts of the melter, the melting part and the clarifying part, are divided by the hot spot. The degree of fitness between hot spots with a cycle formed by the flow determines the effect of clarification then proceeds to the quality of glass; in view of this, the location of the hot spot is important for the float glass process. In the FFDC studied, the hot spot is at a 32.5 m distance from the inlet and a 0.4 m difference from the boundary of Cycles I and II. It can be tolerable in production. Cycle development and the effect of density difference caused by hot spots need a dynamic equilibrium. Excessive differences may bring an obvious concern; that is, the bubble cannot move to the surface of the molten glass, so that clarification loses its effectiveness.

Combined with Figure 6 and Figure 8, less flow velocity has been discovered in FFDCs with traditional structures. It can be analyzed that the branch line that exists divides Cycle III into two parts, which means that the mass flow rate decreases; thus, the acceleration and velocity decrease with the same cross-sectional area as well. That is the basis of better flow stability in FFDC. The temperature distribution is decided by the homogeneity of molten glass. At the same time, the efficiency of convection is better than conduction in this situation. The streamline of FFDC and furnace with a single cooler shows that the branch line causes the molten glass to have more direct flow and more blocks, so that flow is more chaotic than a single one.

When molten glass flows into a cooler of FFDC, the radial spread is shown in the main line and the channel of the branch line. Same as a single line, molten glass in the main line is blocked by a wall and reflux. Except, in the branch line, because of the channel, the streamline performed more chaos. With the combined work of the wall of the cooler, a forward spiral flow is created in the branch line, improving cooling efficacy by increasing the flow heat transfer coefficient and retention time. So, the molten glass has a lower temperature in the branch line, thus benefiting the production of higher-quality glass. Usually, some obliged refrigeration technologies are used in float glass melting furnaces with single coolers to reach the efficacy of cooling like FFDCs. The temperature and flow distributions that the production of glass need is unaffected by the new structure.

Some limitations exist in the numerical simulation model although the basic characteristics of FFDC are investigated. The limitations include the lack of consideration in the flame space, which may cause inaccuracy in edge conditions, and that the more complex mathematical flow model should be used to describe liquid that has a high viscosity. These are recommended for future works.

## 5. Conclusions

Based on the numerical simulation, a float glass melting furnace with a double cooler is declared in temperature distribution and flow characteristics; the following observations are documented:

- Temperature distribution shows a high trend at the middle and low parts at both sides in the temperature of the melter, which is caused by the arrangement of the furnace, and the whole cooler is affected by heat dissipation and heat from the waist, so the temperature decreases along the flow from the inlet of the cooler. Because of the existence of branch lines different from the single cooler, the maximum temperature is located in the waist, which is 1530 K; at the same time, the temperature differences in the surface and bottom at the main and branch lines are 60 K and 43 K, respectively, which indicates that designing the branch cooler makes the stability increase.
- The flow field indicated that there is generally a slow flow in the FFDC. Three cycles similar to the furnace with a single cooler are discovered, which are formed by blocking the bubble boundary, sudden enlargement, and reducing structure combining the double cooler, referred to as the single cooler, which plays a crucial role in inhomogeneity and clarification. Attributed to a more complex channel, the streamline in the furnace is more turbulent so as to well heat transfer and mixed efficiency, which means good quality of glass productions.
- Correspondingly, FFDCs’ temperature and flow characteristics are different from traditional structures and has the potential to improve glass quality. Although the economic efficiency and other operation parameters remain to be proved, it does not prevent the application from being promoted soon and the trend in revolution in glass manufacturing.

## Figures and Tables

**Table 1:** Major size of model.

Part	Length	Width	Height
Melting section	59.4	14	1.2
waist	7.5	4.5	1.15
Main line	20	10	1.15
Branch line	7.5	6.5	1.15
Main outlet	1.02	-	0.3
Branch outlet	1.2	-	0.3

**Table 2:** Material parameters of molten glass.

Parameters	Values	Units
Specific heat capacity	1217	J/kg·K
Density ρ(T)	−0.764 T + 3340	kg/m^3^
Effective thermal conductivity λ(T)	430 − 0.625 T + 0.00025 T^2^	W/m^2^·K
Dynamic viscosity μ(T)	10e^(−2.58+4332/(T−561))^	Pa·s

**Table 3:** Mesh independence verification.

Items	Results before Refinement	Refined by 10%	Refined by—10%	Max Error Value
Minimum temperature (K)	1369.27	1368.94	1351.47	1.30%
Maximum velocity (m/s)	0.027	0.027	0.027	0%
Characteristics of flow field	No changes			-
Temperature distribution	No changes			-

## Data Availability

The original contributions presented in the study are included in the article; further inquiries can be directed to the corresponding authors.

## Glossary

**Symbols**	**Name**	**Units**
E	Total kinetic of the fluid element	J/kg
g_i_	Gravity component in i-direction	m/s^2^
h_j_	Enthalpy of component j	J/kg·K
J_j_	Diffusion flux of component j	-
k_dff_	The effective thermal conductivity	W/m^2^·K
P	Absolute pressure	Pa
R	Distributed resistance	-
r	The equivalent diameter of bubble	m
S_h_	The source term of volumetric heat	-
V_x_, V_y_, V_z_	Velocity component of the molten glass in directions x, y, and z	m/s
v_f_	The velocity of bubble lift	m/s
λ(T)	Thermal conductivity	W/m^2^·K
μ(T)	Dynamic viscosity	Pa·s
Δρ	Density difference in liquid glass and gas in bubble	kg/m^3^
ρ(t)	Density of the molten glass	kg/m^3^
τ	Time	s
